# Draft Genome Sequence of *Propionispora* sp. Strain 2/2-37, a New Xylan-Degrading Bacterium Isolated from a Mesophilic Biogas Reactor

**DOI:** 10.1128/genomeA.00609-16

**Published:** 2016-06-23

**Authors:** Daniela E. Koeck, Irena Maus, Daniel Wibberg, Anika Winkler, Vladimir V. Zverlov, Wolfgang Liebl, Alfred Pühler, Wolfgang H. Schwarz, Andreas Schlüter

**Affiliations:** a Institute for Microbiology, Technische Universität München, Freising, Weihenstephan, Germany; b Institute of Molecular Genetics, Russian Academy of Science, Moscow, Russia; c Center for Biotechnology (CeBiTec), Institute for Genome Research and Systems Biology, Bielefeld University, Bielefeld, Germany

## Abstract

The novel mesophilic bacterial strain *Propionispora* sp. 2/2-37 was isolated from an industrial-scale biogas plant. Comparative 16S rRNA gene sequencing revealed that the isolate constitutes a new subcluster within the order *Selenomonadales*. The 2/2-37 draft genome sequence was established and provides the genetic basis for application of this microorganism in degradation of biomass for bio-fuel production.

## GENOME ANNOUNCEMENT

Detailed knowledge on community members involved in anaerobic digestion (AD) of biomass for biofuel production is considered to be key for process shaping and development of optimization strategies. Therefore, analysis of new AD isolates is pivotal. Genes encoding new lignocellulosic biomass-degrading enzymes for various industrial applications can be discovered in genome sequence information of AD strains.

Strain 2/2-37 was isolated as described previously (1) from an industrial mesophilic one-phase biogas reactor digesting a mixture of maize silage, wheat straw, and manure. The obtained isolate belongs to the genus *Propionispora* within the order *Selenomonadales* (class *Negativicutes*) showing 95% 16S rRNA gene sequence similarity to *Propionispora hippei* DSM 15287^T^ (2). Currently, the strain 2/2-37 cannot be assigned to any validly described family and therefore was classified as belonging to the tentative taxon *Selenomonadales incertae sedis*. Actually, the phylogenetic position of the genus *Propionispora* within the family *Veillonellaceae* is controversial (3). Therefore, a clear taxonomic classification of the isolate 2/2-37 is difficult to specify. *Propionispora* sp. strain 2/2-37 is the first member of the class *Negativicutes* originating from an industrial-scale biogas plant. Similar to its closest relative *Propionispora hippei* DSM 15287^T^, the new isolate forms vibrio-shaped cells, is motile, and develops terminal round spores. Temperature and pH optima for growth are approximately 37°C and pH 6.8, respectively, as described for *Propionispora hippei* (2).

Isolation of genomic DNA for construction of an 8-kb mate-pair sequencing library and sequencing on the Illumina MiSeq system applying the paired-end protocol were accomplished as described previously (4). The sequencing run yielded 7,619,324 reads, accounting for 2,048,864,807 bases of total sequence information. Obtained sequences were *de novo* assembled (5, 6) using the GS *De Novo* Assembler software (version 2.8, Roche). The assembly resulted in formation of 38 scaffolds comprising 84 contigs. Subsequent to *in silico* finishing, 43 contigs remained. The *Propionispora* sp. 2/2-37 draft genome is 4,122,013 bp in size, featuring an average GC content of 45.58%. The software platform GenDB (7) was applied to annotate the *Propionispora* sp. 2/2-37 genome. A total of 3,960 protein coding sequences, 76 tRNA genes, and 1 *rrn* operon were identified.

Analysis and interpretation of the *Propionispora* sp. 2/2-37 genome sequence within GenDB and by means of the carbohydrate-active enzyme database dbCAN (8) revealed 150 genes predicted to encode enzymes that mainly belong to different families of glycoside hydrolases (GH) and glycosyl transferases (GT). Among them are genes presumably facilitating growth of the strain 2/2-37 on a great variety of mono-, di-, and polysaccharides, including cellobiose, sorbitol, xylan, and xylooligosaccharide, indicating involvement of the strain in acidogenesis in the course of AD. Propionic and acetic acid are the major end products of corresponding fermentation processes.

Moreover, genome analysis of the strain *Propionispora* sp. 2/2-37 applying the phage search tool PHAST (9) revealed insertions of three prophages having sizes of approximately 32 to 54 kb each. Presence of prophage regions in the *Propionispora* sp. 2/2-37 genome exemplarily illustrates that biogas community members have to deal with phage infections, which also may impact the whole biogas production process.

### Nucleotide sequence accession numbers.

This whole-genome shotgun project has been deposited in the EMBL/GenBank database (EBI, NCBI) under the accession numbers CYSP01000001 through CYSP01000043. The strain is available from the Leibniz Institute “German Collection of Microorganisms and Cell Cultures” (DSMZ, Braunschweig, Germany) under the accession number DSM 100628.
